# Supplementary material for: A Novel Phantom for Standardized Microcalcification Detection Developed Using a Crystalline Growth System
Source: Tomography. 2025 Feb 27;11(3):25. doi: 10.3390/tomography11030025 (PMC11945459; doi:10.3390/tomography11030025)
Supplement: Supplementary file 1 [file tomography-11-00025-s001.zip › tomography-3384401-supplementary.pdf]

Supplemental Document

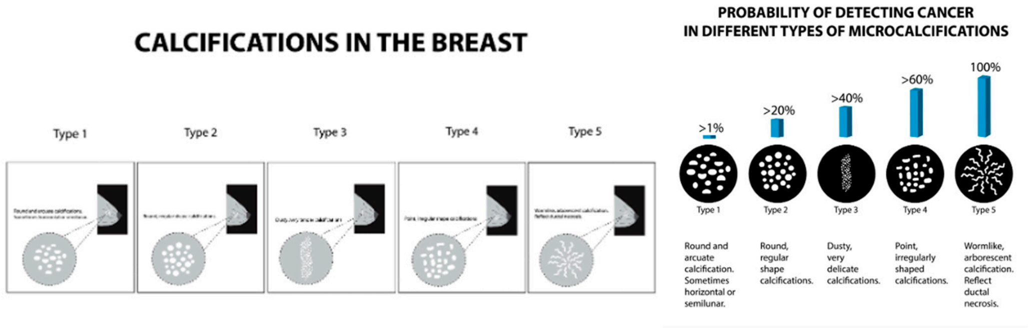

Figure S.1. Calcifications can appear in various complex shapes, and the grade/severity of the potential for cancer and whether it is benign or malignant can be determined. Various shapes include appearances of “powdery,” “cloud-like,” “cotton-like," linear, inhomogeneous, or spiculated. We can classify microcalcifications broadly as 1) coarse heterogeneous, 2) amorphous, 3) fine pleomorphic, or 4) fine linear or fine-linear branching. Calcification assessment on DBT is challenging yet crucial as a diagnostic tool for breast radiologists to determine patient care pathways.

S.1 Detection and Diagnostic Challenges of Calcifications in Breast Cancer

Calcifications, often the earliest indicator of ductal carcinoma in situ (DCIS) and early-stage invasive breast cancer, are detected in screening mammograms and serve as a critical diagnostic marker for breast cancer [23]. In many cases, especially for younger women with dense breast tissue, detecting these calcifications is challenging, as dense tissue can obscure minor abnormalities. Mammographic findings classified as BI-RADS category 0 on screening examination, as seen in Figure S.1, prompt a recall for further evaluation, which may include additional imaging, ultrasound, and potentially invasive procedures like fine needle aspiration (FNA) or core needle biopsy (CNB) [24].

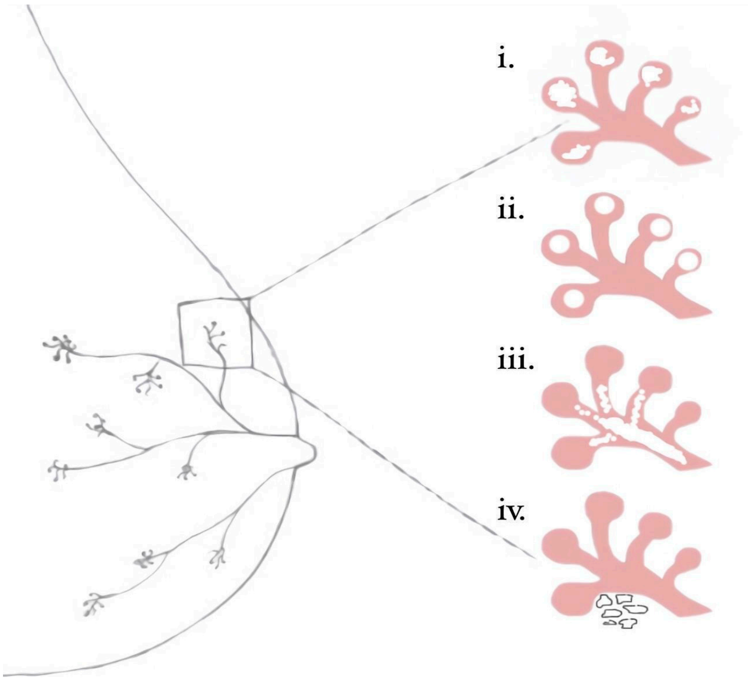

Figure S.2. Breast mammary duct with illustrated benign and malignant calcification patterns as outlined in BI-RADS classifications: I) amorphous II) milk of calcium III) fine linear branching, IV) course heterogeneous.

S.2 Physiological and Diagnostic Features of Cancer Subtypes Targeted for Improved Visualization under DBT.

One of the earliest signs of some types of breast cancer is microcalcifications, which are commonly associated with DCIS [23], and thus, microcalcifications are one major imaging feature assessed in breast mammography. Different breast pathologies can give rise to different patterns of calcifications on mammography. Examples of benign breast pathology include simple cysts, fat necrosis, fibroadenomas, and ductal ectasia. Benign calcifications can appear as eggshell-like, popcorn-like, and large rod-like calcifications [10,13]. The morphologic appearance of microcalcifications on mammography can help determine the likelihood of breast malignancy (Figure S.2). The complexity of visualizing and diagnosing breast calcifications highlights the need for standardized mammography technologies, which would enable radiologists to achieve greater diagnostic accuracy. Early detection of breast cancer in women can significantly reduce both morbidity and mortality rates. Screening mammography exams play a crucial role in this early detection process [25]. Variations in the ability of mammography systems to visualize microcalcifications can lead to inconsistent interpretations and patient management. Providing physicians with consistently high-quality images across technologies, vendors, and institutions is crucial for accurate diagnoses, efficient workflows, and optimal patient care.

S.3. Ensuring Compliance and Advancing Technology in Breast Tomosynthesis

Obtaining high-quality images of breast calcifications is a critical responsibility of a mammography center and the healthcare workers at that institution, and thus, is of crucial importance to mammography scanner manufacturers. Further, the provision of high-quality patient care and safety standards requires that mammography systems comply with rigorous guidelines. These constraints typically cover a range of multifactorial competing objectives that include image quality, radiation dose, equipment performance, and quality control procedures [5]. Thus, compliance with standards is intended to

increase the potential for consistency in breast screening exams [25]. Ensuring that mammography machines comply with established guidelines, such as those specified by the FDA, imposes an immense burden and responsibility on manufacturers, and all manufacturers invest significant resources for testing, evaluation of compliance, and documentation throughout the development of medical scanners. Detecting and characterizing microcalcifications is just one among many design goals in mammography imaging and is an element that can easily be overlooked. Incorporating our advanced phantom model in mammography design promises to significantly improve the uniformity in interpreting calcifications. Standardization is achieved by using a real target, such as a phantom that mimics the conditions radiologists encounter in actual clinical situations, as discussed in our work. This will empower healthcare professionals to assess calcifications more consistently and help reduce the variation in patient recall rates across imaging centers. This approach will streamline patient care, alleviate patient anxiety, and optimize resource use by minimizing unnecessary recalls and ensuring patients are only called back when necessary. Ultimately, our phantom design aims to enhance diagnostic accuracy, leading to more effective and patient-centered breast cancer screening protocols. Test phantoms, such as the sets we propose, will enhance design processes and increase confidence in imaging the types of calcifications seen both during the training of mammographers and in real-world conditions encountered by radiologists.

S.4 Detector Differences and Similarities between Manufacturers

Detector design varies among DBT manufacturers, as shown in Table 1. For instance, both cesium iodide (CsI) with amorphous silicon (a-Si) and amorphous selenium (a-Se) present distinct advantages. CsI with a-Si provides higher detective quantum efficiency (DQE), minimal light loss, and a wide dynamic range, making it versatile for different imaging conditions [26]. In contrast, a-Se allows for direct conversion, resulting in sharper images and higher spatial resolution, which is crucial for detecting small calcifications and fine details. Practical considerations, such as cost, availability, and existing infrastructure, also influence the choice of detector technology.

S.5 Reconstruction of Mammographic Images: complexity for microcalcifications in DBT

Addressing the reconstruction of mammographic microcalcifications through tomosynthesis presents an array of design challenges that require innovative solutions to achieve optimal outcomes. Manufacturers are tasked with employing diverse correction methods to manage noise levels, ensure edge quality, maintain microcalcification visibility, and address out-of-slice artifacts. For instance, Abdurahman et al. utilize transformations for each projection that can be combined from different slice values, although this may impact integrated spatial resolution [27]. This reconstruction design can challenge capturing higher spatial resolution structures such as calcifications. However, reconstruction methods operated on slices can be generated using a statistical artifact reduction (SAR) method followed by inversion modulation transfer function (MTF) and applying empirical spectral and spatial thickness filters, providing promising avenues for advancement to improve complex reconstruction. Furthermore, techniques to manage out-of-slice artifacts are critical for enhancing image quality and diagnostic accuracy. These artifacts, characterized by the blurring or ghosting of structures, not in the plane of interest but appearing in the reconstructed slice, require careful handling. Approaches like super-resolution and regression of structures modeling can enhance performance, with each manufacturer employing various methods to address reconstruction quality. Overall, the ongoing innovative efforts and promising methodologies being explored in this field encourage confidence in the future of mammographic microcalcification reconstruction through tomosynthesis. However, ongoing innovations vary greatly between vendors which can lead to standardization

challenges if not given appropriate targets that can be designed against, such as phantoms.

S.6 Filters used in mammographic DBT

In DBT, various filters optimize image quality and minimize radiation dose, and different vendors implement them in distinct ways. These filters include rhodium (Rh), aluminum (Al), silver (Ag), and molybdenum (Mo). Rh is often used because it effectively filters out lower-energy X-rays, helping to reduce patient dose while maintaining image quality and improving contrast, especially in thicker or denser breasts. Al is another less expensive filter that balances image quality and radiation dose and is one of the primary materials used in current mammography scanners. Additionally, Ag can sometimes be utilized in systems to enhance contrast and improve visualization of breast tissue, while Mo filters, although less common, are also used [5]. In X-ray systems, the focusing cup concentrates the electron beam toward the focal spot on the anode. The focusing cup is a negatively charged depression on the cathode side of the X-ray tube and is typically made of materials chosen for their ability to withstand high temperatures and effectively focus the electron beam. In the focusing cup, mammography device vendors sometimes have one or two filaments that produce focal spots with nominal sizes of 0.3 mm and 0.1 mm. Focal spots of 0.1 mm are used for magnification mammography, achieving high spatial resolution by minimizing geometric blurring [28].
